# Molecular Architectures Derived from Metal Ions and the Flexible 3,3′-Bipyridine Ligand: Unexpected Dimer with Hg(II)

**DOI:** 10.1155/2010/169054

**Published:** 2010-05-30

**Authors:** Anupam Khutia, Pablo J. Sanz Miguel, Bernhard Lippert

**Affiliations:** Fakultät Chemie, Technische Universität Dortmund, 44221 Dortmund, Germany

## Abstract

The flexible ditopic ligand 3,3′-bipyridine (3,3′-bpy) has been reacted with a series of transition metal species (Ag^+^, Hg^2+^, cis-a_2_M^2+^ (a = NH_3_ or a_2_ = en; M = Pt, Pd), trans-a_2_Pt^2+^ (a = NH_3_)) in an attempt to produce discrete cyclic constructs. While Ag^+^ gave a polymeric structure {[Ag(3,3′-bpy)](ClO_4_) · H_2_O}_*n*_ (**1**), with all other metal entities cyclic structures were formed. Interestingly, Hg(CH_3_COO)_2_ produced a dinuclear complex [Hg(3,3′-bpy)(CH_3_COO)_2_]_2_ · 3H_2_O (**2**), in which the two 3,3′-bpy ligands adopt a cis-orientation of the coordinating pyridyl entities. With cis-(NH_3_)_2_Pt^2+^, a cyclic complex **4** was isolated in crystalline form which, according to HRMS, is a trimer. With trans-(NH_3_)_2_Pt^2+^, different species are formed according to ^1^H NMR spectroscopy, the nature of which was not established.

## 1. Introduction

**Scheme 1:**
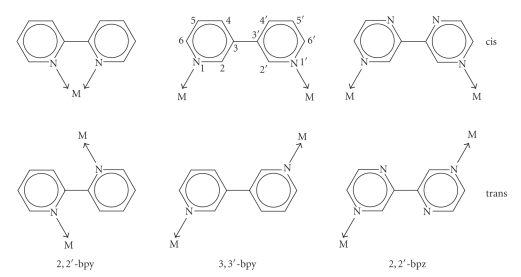
cis- and trans-orientation of pyridine and pyrazine rings in 2,2′-bpy, 3,3′-bpy, and 2,2′-bpz.

The “molecular library” concept has proven highly efficient in designing discrete supramolecular metal complexes by combining di- or multitopic metal entities with rigid di- or multitopic ligands [1, 2]. It is less straightforward if ligands are flexible and can adopt, in principle, different rotamer states. In its simplest form, this is the case when two N-heterocyclic ligands are connected via a C–C bond. Examples are, among others, 2,2′-bipyridine (2,2′-bpy), 3,3′-bipyridine (3,3′-bpy) and, 2,2′-bipyrazine (2,2′-bpz) (Scheme 1). While 2,2′-bpy, in the overwhelming number of structures, acts as a chelating ligand with the two ring N atoms in a cis-orientation, there are also rare cases of 2,2′-bpy adopting a bridging mode, hence being in a transconfiguration or half-way between cis and trans- [3]. It depends on the conformation of the ligand and the geometry of the metal, what kind of construct/s is/are formed. With 2,2′-bpz, we have studied this question in more detail and have characterized a number of discrete molecular entities, which include a flat triangular structure, 3D triangular entities of different shapes (prism, vase), as well as a tetranuclear open box [4, 5]. In all these cases the N4/N4′ positions are involved in metal coordination, occasionally complemented by addition of metal chelation via N1/N1′, and influenced by counter anions.

**Figure 1:**
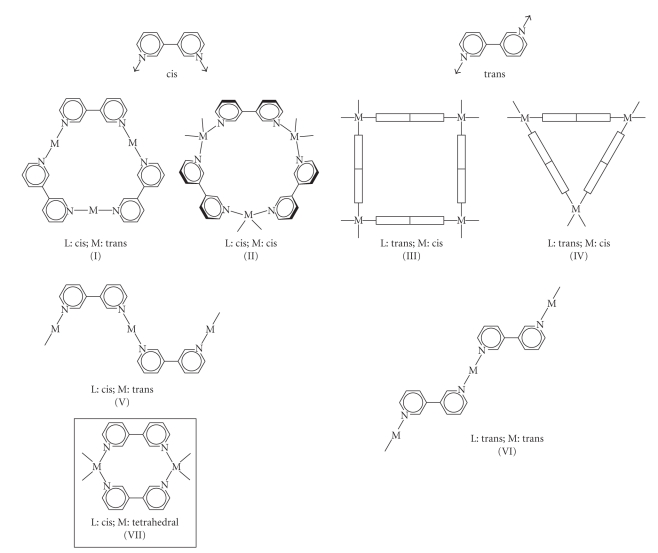
Feasible discrete (I–IV) and polymeric (V, VI) structures of 3,3′-bpy metal complexes, and novel dinuclear complex (VII) observed in the Hg^II^ complex **2**.

In principle, 3,3′-bipyridine (3,3′-bpy) metal complexes should be able to reveal analogous topologies as 2,2′-bpz, with the advantage of higher basicities of the N donor atoms (Figure 1). There are several reports in the literature on polymeric structures containing cis- [6] and in particular trans-arranged 3,3′-bpy ligands [7], yet none with a discrete molecular metallacycle. The only related examples are those of trinuclear cycles containing three cis-a_2_M^II^ units (a_2_ = diamine; M = Pd or Pt) and three 4,7-phenanthroline ligands, which can be considered rigid analogous of 3,3′-bpy ligands with the two pyridine entities fixed in a cis-orientation [8, 9]. Our interest in discrete cationic metallacycles stems, among others, from their potential of interacting noncovalently with DNA [10] or particular DNA secondary structures such as DNA quadruplexes [11], as well as their ability to act as hosts for anions [12, 13]. In the present study, we have employed different transition metal ions and metal entities which previously have been shown by others and ourselves to produce discrete cyclic complexes, namely Ag(I), Hg(II), enPd(II), cis-(NH_3_)_2_Pt(II) as well as trans-a_2_Pt(II) (a = NH_3_) [14–16].

## 2. Experimental

### 2.1. Synthesis Procedures

AgClO_4_ and Hg(CH_3_COO)_2_ were of commercial origin. 3,3′-bpy [17], PdCl_2_(en) [18], cis-PtCl_2_(NH_3_)_2 _[19], and trans-PtCl_2_(NH_3_)_2_ [20] were prepared according to known literature procedures.

**{[Ag(3,3′-bpy)] (ClO_4_)·H_2_O}_*n*_ (1)**

To a solution of 3,3′-bpy (15.6 mg, 0.1 mmoL) in water (3 mL), an aqueous solution (2 mL) of AgClO_4_ (20.7 mg, 0.1 mmoL) was added. The white precipitate which formed immediately was centrifuged off and recrystallized from water (4 mL, 40°C). Colorless crystals were obtained after 2 d at room temperature. Yield: 30.5 mg (80%). Anal. Calcd (%) for C_10_H_10_AgClN_2_O_5_: C, 31.5; H, 2.6; N, 7.3. Found: C, 31.4; H, 2.6; N, 7.5.

**[Hg(3,3′-bpy)(CH_3_COO)_2_]_2_·3H_2_O (2)**

An aqueous solution (4 mL) of 3,3′-bpy (15.6 mg, 0.1 mmoL) and Hg(CH_3_COO)_2_ (31.9 mg, 0.1 mmoL) was stirred at room temperature for 12 h. The solution is filtered and kept at room temperature. After 3 d, colorless crystals were obtained. Yield: 37.1 mg (74%). **2** was characterized by X-ray analysis.

**[{Pd(en)(3,3′-bpy)}(NO_3_)_2_·H_2_O]_*n*_ (3)**

An aqueous suspension (15 mL) of PdCl_2_(en) (47.4 mg, 0.2 mmoL) and AgNO_3_ (68 mg, 0.4 mmoL) was stirred in dark for 12 h. The resultant AgCl precipitate was filtered off and 3,3′-bpy (31.2 mg, 0.2 mmoL) was added to the filtrate. The solution was stirred at 40°C for 1 day and then concentrated to a volume of 4 mL by rotary evaporator. The solution was filtered and kept at room temperature. After 4 d, light yellow powder was recovered. Yield: 61 mg (66%). Anal. Calcd (%) for (C_12_H_18_N_6_O_7_Pd)*_n_* (1-hydrate): C, 31.0; H, 3.9; N, 18.1. Found: C, 30.8; H, 4.0; N, 18.0.

**cis-[{Pt(NH_3_)_ 2 _(3,3′-bpy)}(PF_6_)_2_·H_2_O]_*n*_ (4)**

An aqueous suspension (20 mL) of cis-PtCl_2_(NH_3_)_2_ (60 mg, 0.2 mmoL) and AgNO_3_ (68 mg, 0.4 mmoL) was stirred in dark for 12 h. The resultant AgCl precipitate was filtered off and 3,3′-bpy (31.2 mg, 0.2 mmoL) was added to the filtrate. The solution was stirred at 60°C for 3 d, then solid NH_4_PF_6_ (65.2 mg, 0.4 mmoL) was added to it and the solution was stirred at 60°C for another day. The solution was concentrated to a volume of 5 mL (pD = 3.20) and kept in an open beaker at 4°C. After 5 d, colorless crystals were obtained. According to MS, **4** represents a cyclic trimer, hence *n* = 3. Yield: 73 mg (54%). Anal. Calcd (%) for C_30_H_48_N_12_O_3_P_6_F_36_Pt_3_: C, 17.3; H, 2.3; N, 8.1. Found: C, 17.5; H, 2.6; N, 7.9.

### 2.2. X-Ray Crystal Structure Determination

X-ray crystal data for **1** and **2** (Table 1) were recorded at 150 K with an Xcalibur diffractometer equipped with an area detector and graphite monochromated Mo K*α* radiation (0.71073 Å). Data reduction was done with the CrysAlisPro software [21]. Both structures were solved by direct methods and refined by full-matrix least-squares methods based on *F* ^2^ using SHELXL-97 [22]. All nonhydrogen atoms were refined anisotropically. Hydrogen atoms (including water molecules) were positioned geometrically and refined with isotropic displacement parameters according to the riding model. All calculations were performed using the SHELXL-97 and WinGX programs [22, 23]. CCDC 763713 and 763714 contain the crystallographic data for compounds **1** and **2**.

### 2.3. Instruments

Elemental (C, H, N) analysis data were obtained on a Leco CHNS-932 instrument. The ^1^H NMR spectra were recorded in D_2_O with tetramethylammonium chloride (TMA) and sodium-3-(trimethylsilyl)-1-propanesulfonate (TSP) as internal reference, on Bruker AC 200 and Bruker AC 300 spectrometers.

### 2.4. Electrospray Mass Spectrometry

The mass spectrum of **4** was recorded with an LTQ orbitrap (high resolution mass spectrometer) coupled to an Accela HPLC-system (consisting of Accela pump, Accela autosampler, and Accela PDA detector), from Thermo Electron. The parameters for HPLC were as follows: (i) Eluent A (0.1% formic acid in H_2_O) and eluent B (0.1% formic acid in acetonitrile) with mobile phase consisting of 50% A and 50% B, (ii) Flow rate 250 *μ*L/min, (iii) injection volume 5 *μ*L, (iv) scan of wavelength range from 200 to 600 nm. The parameters for MS were as follows: (i) ionisation mode ESI (electrospray ionization), (ii) source voltage 3.8 kV, Capillary voltage 41 V, Capillary temperature 275°C, tube lens voltage 140 V, (iii) scanned mass range 150 m/z to 2000 m/z with resolution set to 60000. Analysis was done by flow injection (without any column).

### 2.5. Determination of pK_a_ Values

The pK_*a*_ values of 3,3′-bpy ligand were determined by evaluating the changes in chemical shifts of bipyridine protons at different pD values. pD values were measured by use of a glass electrode and addition of 0.4 units to the uncorrected pH meter reading (pH*). The graphs (chemical shifts versus pD) were evaluated with a nonlinear least-squares fit according to Newton-Gauss method [24] and the acidity constants (calculated for D_2_O) were converted to values valid for H_2_O [25].

## 3. Results and Discussion

**^1^H NMR Spectra of 3, 3′-Bipyridine**

**Figure 2:**
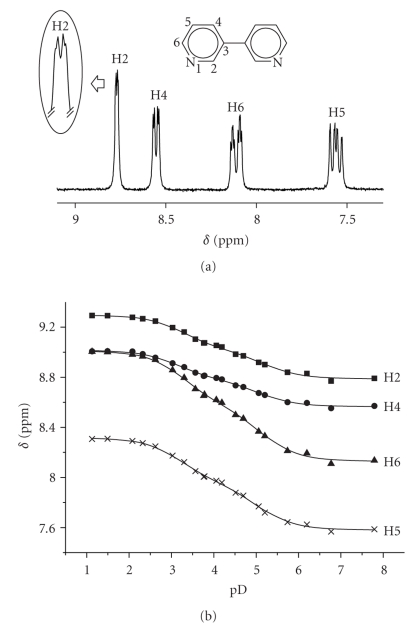
(a) Low field section of ^1^H NMR spectrum of 3,3′-bpy (D_2_O, pD = 6.8) and pD dependence of individual resonances, focusing splitting of the H2 resonances. (b) pD dependence of H2, H4, H6, and H8 resonances of free 3,3′-bipyridine.

Figure 2 displays a typical ^1^H NMR spectrum of the free ligand at pD 6.8. The individual resonances show the expected coupling patterns [17]. In the D_2_O spectrum, all resonances show splitting due to long-range coupling. For example, the H2 signal is split into a doublet due to coupling with H4 (1.5 Hz) and additionally displays coupling with H5 (0.7 Hz). Upon protonation, all resonances are downfield shifted, with H6 affected most. pK_*a*_ values for [3,3′-bpyH]^+^ and [3,3′-bpyH_2_]^2+^, as determined by pD dependent ^1^H NMR spectroscopy, are 4.58 ± 0.1 and 2.71 ± 0.1 (values converted to H_2_O), respectively. These values compare with 4.3 and *ca.* 0.3 for 2,2′-bpy, and 0.45 and –1.35 for 2,2′-bpz, and reflect the higher basicity of 3,3′-bpy as compared to two other ligands.

^1^H NMR resonances of 3,3′-bpy in D_2_O display a moderate sensitivity on concentration, which is consistent with intermolecular stacking. For example, when going from 0.0125 M to 0.125 M, upfield shifts are 0.06 ppm (H2), 0.03 ppm (H4), 0.05 ppm (H6), and 0.04 ppm (H5).

**Ag^+^ and Hg^2+^ Coordination**

Addition of Ag^+^ ions to an aqueous solution of 3,3′-bpy in D_2_O expectedly does not reveal resonances due to individual species, but rather gives only averaged signals of the free ligand and the various Ag complexes as a consequence of fast exchange.

A similar situation applies to mixtures of 3,3′-bpy and Hg(II) acetate. The spectrum of the dinuclear Hg(II) complex **2** has its ^1^H resonances (*δ*, ppm; D_2_O, pD 5.2) at 8.97, 8.73, 8.44, and 7.88 as well as 2.02 (acetate). No coupling of any of the 3,3′-bpy resonances with the ^199^Hg isotope is observed as in a previously reported case [26], and a comparison of the shifts of **2** with those of the free ligand at the same pD (downfields shifts of H2, 0.06 ppm; H4, 0.06 ppm; H6, 0.09 ppm; H5, 0.13 ppm) does not permit any conclusions regarding the bonding situation in solution.

**Figure 3:**
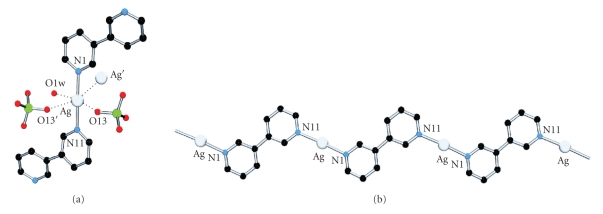
(a) Detail of the coordination sphere of the silver atom in **1**. (b) Polymeric motif between Ag1 and 3,3′-bpy bridging ligands in **1**.

**Figure 4:**
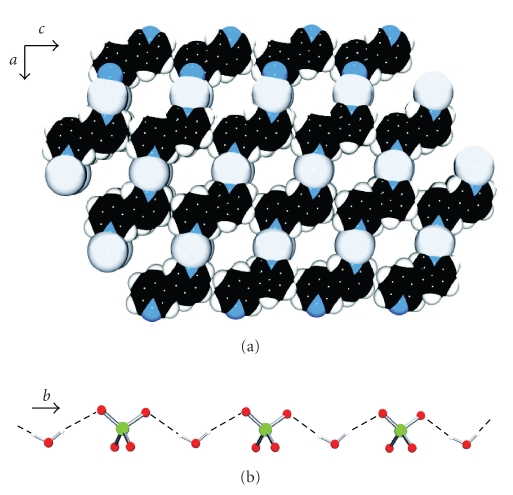
(a) Section of the packing pattern in **1** (excluding H_2_O and ClO_4_^−^), including voids along the *b* direction. (b) Water-perchlorate hydrogen bonded polymer inserted along the packing tunnels of **1**.

The crystal structure of {[Ag(3,3′-bpy)](ClO_4_) · H_2_O}_*n*_ (**1**) reveals a polymeric structure rather than a discrete cyclic structure as we had hoped for. The silver atom (Ag1) shows a distorted octahedral coordination sphere (Figure 3(a)), with two 3,3′-bpy ligands at the apical positions (Ag1-N1, 2.181(3) Å; Ag1-N11, 2.189(3) Å). The equatorial coordination is completed by a water molecule (Ag1-O1w, 2.722(3) Å), two perchlorate counter anions (Ag1-O13, 2.773(4) Å; Ag1-O13′, 2.861(4) Å), and an argentophilic interaction [27, 28] with a neighbor silver atom (Ag ⋯ Ag, 3.3751(8) Å). Angles and distances involving the coordination sphere of Ag1 are listed in Table 2. The polymeric structure is assembled by coordination of additional silver units to the bridging 3,3′-bpy ligands of the apical positions, with a –Ag–[N11-3,3′-bpy-N21]–Ag– basic motif, which extends along the [1 0 1] direction (Figure 3(b)). The 3,3′-bpy ligands adopt transconformations with a twist angle of 27.9(1)° between pyridine halves. The dihedral angle between two pyridyl rings coordinated to Ag1 is 7.2(1)°. The crystal packing is based on *π* − *π* stacking and argentophilic interactions between polymer strands. An upper view of the *ac* plane evidences the presence of voids in the structure (Figure 4(a)). They are essentially rectangular tunnels along the *b* axis, which house two sets of hydrogen bond-based perchlorate-water polymers. These polymers are built by connecting water molecules of crystallization and perchlorate anions. Each O1w forms two hydrogen bonds with two perchlorate anions: ⋯ O14-Cl1-O12 ⋯ (H1w)O1w(H2w) ⋯ O14-Cl1-O12 ⋯ (Figure 4(b)). Distances and angles involving O1w are O1w ⋯ O12, 2.969(5) Å; O1w ⋯ O14, 2.886(5) Å; O12 ⋯ O1w ⋯ O14, 119.5(2)°.

**Figure 5:**
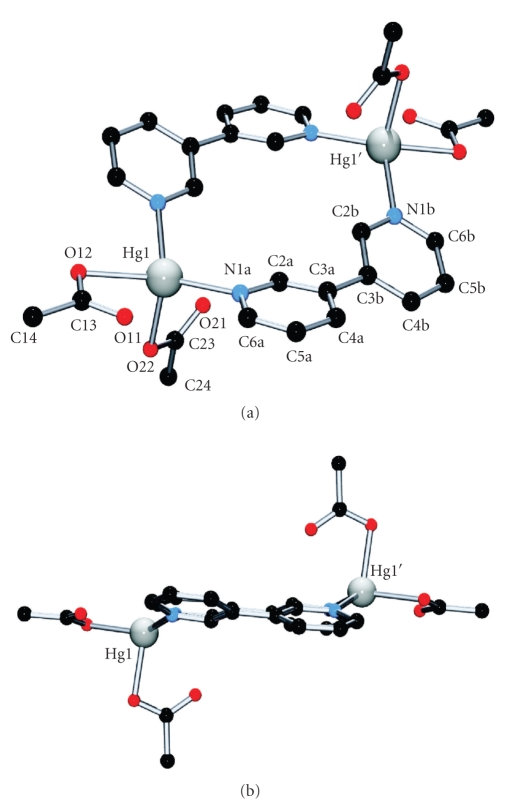
(a) View of **2** with atom numbering scheme. (b) Side view of **2**, evidencing a boat conformation of the mercury atoms with respect to the 3,3′-bpy ligands.

The crystal structure of the dinuclear species [Hg(3,3'-bpy)(CH_3_COO)_2_]_2_· 3H_2_O (**2**) is given in Figure 5. Unlike in **1**, in **2** the 3,3′-bpy ligands adopt a cis-conformation of the two pyridyl rings, with a twist angle of 30.4(2)°, and act as bridges between two mercury centers. The coordination geometry of the Hg ion (Table 3) is distorted tetrahedral, enclosing two 3,3′-bpy entities (Hg1-N1a, 2.274(3) Å; Hg1-N1b, 2.263(3) Å), and two chelating/semichelating acetates (Hg1-O11, 2.490(3) Å; Hg1-O12, 2.392(3) Å; and Hg1-O21, 2.286(3) Å; Hg1-O22, 2.762(3) Å). Selected distances and angles around mercury are listed in Table 3. Both 3,3′-bpy ligands and their bonded mercury atoms are almost coplanar with a tendency towards a boat conformation (distance from Hg1 to the plane defined by N1a, N1b, N1a′, N1b′ is 0.58 Å).

The disposition of the acetate ligands is worthy to be discussed in more detail. Both ligands form a dihedral angle of 79.23(16)° with each other. The ligand containing O11,O12 is roughly coplanar with the pyridyl rings (7.27(27)°, 23.28(23)°), whereas the ligand A2 (with O21,O22) is roughly perpendicular (78.33(15), 72.81(14)°). Both are asymmetrically coordinated to Hg1, displaying significant longer bond distances of those oxygen atoms involved in hydrogen bonding: O1w ⋯ O11, 2.802(5) Å (Hg1-O11, 2.490(3) Å versus Hg1-O12, 2.392(3) Å) and O1w ⋯ O22, 2.757(4) Å (Hg1-O21, 2.286(3) Å versus Hg1-O22, 2.762(3) Å). Further hydrogen bonding includes a twofold O1w ⋯ O2w (2.785(10) Å) connection. Besides hydrogen bonding, the crystal packing includes *π* − *π*- and anion–*π*-interactions. N1a-pyridyl rings are pairwise *π* − *π* stacked (3.5 Å), and both rings are involved in an additional anion–*π*-interaction with O11 (O11 ⋯ centroid, 3.47 Å). Considering the latter, the formation of staggered rows is observed, in which each molecule displays four anion–*π*-interactions with neighbor molecules. Rows are interconnected by *π* − *π*-stacking and hydrogen bonding.

**Complexes with enPd^II^ and cis-(NH_3_)_2_Pt^II^**

**Figure 6:**
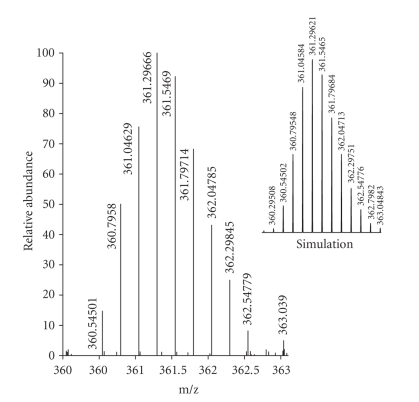
HRMS spectrum of complex **4**: Observed and calculated pattern for [M–(PF_6_)_4_]^4+^.

Reactions of 3,3′-bpy with [Pd(en)(H_2_O)_2_](NO_3_)_2_ and cis-[Pt(NH_3_)_2_(H_2_O)_2_](NO_3_)_2_ (1 : 1 ratio) give products of 1 : 1 stoichiometry [{Pd(en)(3,3′-bpy)}(NO_3_)_2_]_n_ (**3**) and cis-[{Pt(NH_3_)_2_(3,3′-bpy)}(PF_6_)_2_]_n_ (**4**) which, according to ^1^H NMR spectroscopy, are pure materials. Only single sets of 3,3′-bpy resonances are observed in both compounds, indicating that both compounds must be cyclic. Chemical shifts (*δ*, ppm; D_2_O, TMA as internal reference) are as follows: **3**, 9.15, 8.84, 8.27, 7.69 ppm (3,3′-bpy) and 2.98 (en); **4**, 8.99, 8.95, 8.23, 7.67 (3,3′-bpy). When TSP was used as a reference, shifts differed by 0.1 ppm (**3**) and 0.09 ppm (**4**), suggesting that the TSP anion interacts with the cations of **3** and **4** [29]. Although **4** was isolated in microcrystalline form, an X-ray structure determination proved impossible. The high resolution MS of a sample of **4** was carried out and confirmed a triangular structure (see (II) or (IV) in Figure 1). The mass spectrum displayed peaks due to [M–(PF_6_)]^+^: 1880.08114 (calcd. 1880.07904), (M–(PF_6_)_2_]^2+^: 867.55844 (calcd. 867.55828), and [M–(PF_6_)_4_]^4+^: 361.29666 (calcd. 361.29621). The HRMS spectrum of [M–(PF_6_)_4_]^4+^ is given in Figure 6 and compared with the simulated spectrum.

**Reaction with trans-[Pt(NH_3_)_2_(H_2_O)_2_]^2+^**

**Figure 7:**
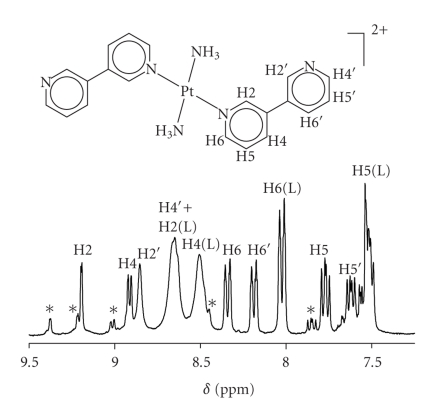
^1^H NMR spectrum of mixture of trans-[Pt(NH_3_)_2_(D_2_O)_2_]^2+^ and 3,3′-bpy (L) in ratio 1 : 10 after 2 d, 50°C, D_2_O, pD = 6.65. Main resonances are assigned to 1 : 2 complex and free ligand; minor resonances (*) are not assigned.

Reaction of 3,3′-bpy with trans-[Pt(NH_3_)_2_(D_2_O)_2_](NO_3_)_2_ was carried out with different ratios between 3,3′-bpy and the Pt species (10 : 1, 2 : 1, 1 : 1, 1 : 10) on the ^1^H NMR scale in D_2_O. Without exception, the spectra displayed time-dependent changes, but within 2-3 d at 50°C, constant spectra were obtained. Even then, however, resonances due to multiple products were present. In the case of a large excess of ligand over Pt (10 : 1), the spectrum reveals the presence of a major species attributed to trans-[Pt(NH_3_)_2_(3,3′-bpy)_2_]^2+^ and excess 3,3′-bpy (Figure 7). The resonances of the free 3,3′-bpy (L) were unambiguously identified by adding solid 3,3′-bpy to the NMR sample. The two sets of pyridine resonances of the coordinated 3,3′-bpy ligands of the 1 : 2 complex are assigned on the basis of their relative intensities. What strikes is that the H2 and H4 resonances of the free ligand are very much broadened (cf. Figure 2(a)) and that H2, H4, and H6 are upfield shifted by *ca.* 0.2, 0.08, and 0.14 ppm, respectively. As these shifts cannot be interpreted with a pD effect, we propose that the presence of the 1 : 2-Pt complex has an effect on the rotamer equilibrium of the free ligand. Consistent with this proposal, the two resonances closest to the C3–C3 bond, hence H2 and H4, become quite broad. Stacking interactions between free and coordinated 3,3′-bpy could possibly account for this feature.

The ^1^H NMR spectrum of a 1 : 1 mixture of trans-[Pt(NH_3_)_2_(D_2_O)_2_]^2+^ and 3,3′-bpy displays four H2 (H2′) singlets of different relative intensities at lowest field, and at least for the H6 (H6′) resonances also four components can be differentiated. Free 3,3′-bpy is not detectable. It is obvious that the self-assembly process of trans-(NH_3_)_2_Pt^II^ and 3,3′-bpy does not lead to a preferred single product, unlike in the case of enPd^II^ and cis-(NH_3_)_2_Pt^II^.

## 4. Summary

The flexible ditopic ligand 3,3′-bipyridine forms with Hg(CH_3_COO)_2_ and cis-[Pt(NH_3_)_2_(H_2_O)_2_](PF_6_)_2_ discrete di- and trinuclear cycles **2** and **4**, respectively. The solid state structure of the Hg(II) complex **2** is unique in that it represents the smallest possible entity of any cyclic complex. It appears that the opening of the N1a-Hg-N1b angle to *ca.* 115° allows the dinuclear to be formed. A similar structure, with the two 3,3′-bpy ligands approximately coplanar, is not to be expected for cis-a_2_Pt^II^ with its 90° bonding angle. Consequently, **4** is a cyclic trinuclear compound. On the NMR time scale, **2** is kinetically labile in aqueous solution, but **4** is inert. We plan to further study **4** with regard to its host-guest chemistry and its noncovalent interaction with DNA.

## Figures and Tables

**Table 1:** Crystallographic data for compounds {[Ag(3,3′-bpy)](ClO_4_) · H_2_O}_*n*_ **(1)** and [Hg(3,3′-bpy)(CH_3_COO)_2_]_2_· 3H_2_O **(2)**.

	**1**	**2**
Formula	C_10_H_10_Ag_1_Cl_1_N_2_O_5_	C_28_H_34_Hg_2_N_4_O_11_
Formula weight (g moL^−1^)	381.52	1003.77
Crystal color and habit	colorless prisms	colorless prisms
Crystal size (mm)	0.20 × 0.20 × 0.10	0.15 × 0.10 × 0.05
Crystal system	monoclinic	triclinic
Space group	*P*2_1_/c	*P*-1
*a* (Å)	9.7606(10)	8.5635(5)
*b* (Å)	7.3145(8)	9.2096(6)
*c* (Å)	19.572(2)	11.3262(6)
*α* (°)	90	74.746(5)
*β* (°)	119.148(9)	84.183(4)
*γ* (°)	90	63.221(6)
*V* (Å^3^)	1220.4(2)	769.21(8)
*Z*	4	1
*D*_calcd._ (g cm^−3^)	2.077	2.167
*F* (000)	752	478
*μ* (mm^−1^)	1.888	10.034
No. reflections collected	2333	3572
No. reflections observed	1557	2969
*R*_int _	0.0317	0.0359
No. parameters refined	172	208
*R* [*I* > 2*σ*(*I*)]	0.0331	0.0270
*wR* (all reflections)	0.0572	0.0453
Goodness-of-fit (GOF)	1.041	0.911
Δ*ρ*_max _and Δ*ρ*_min _(*e* Å^−3^)	0.941 and –0.517	1.078 and –1.283

GOF = [Σ*w*(*F*_*o*_^2^−*F*_*c*_^2^)^2^/(*N *_o_−*N_*ν*_*)]^1/2^; *R* = Σ||*F*_*o*_|−|*F*_*c*_||/Σ|*F*_*o*_|; *wR* = [Σ(*w*(*F*_*o*_^2^−*F*_*c*_^2^)^2^)/Σ*w*(*F*_*o*_^2^)^2^]^1/2^.

**Table 2:** Selected bond distances (Å) and angles (*º*) for compound **1**.

Ag1-N1, 2.181(3)	N1-Ag1-N11, 174.37(13)	N11-Ag1-Ag1′, 76.06(9)
Ag1-N11, 2.189(3)	O13-Ag1-Ag1, 157.52(8)	N11-Ag1-O13, 87.31(11)
Ag1-Ag1, 3.3751(8)	O1w-Ag1-O13, 168.17(10)	N11-Ag1-O13′, 92.86(11)
Ag1-O1w, 2.722(3)	N1-Ag1-Ag1′, 108.03(9)	N11-Ag1 O1W 95.33(11)
Ag1-O13, 2.773(4)	N1-Ag1-O13, 84.58(11)	Ag1′-Ag1-O1w, 78.60(7)
Ag1-O13′, 2.861(4)	N1-Ag1-O13′, 87.48(11)	O1w-Ag1-O13, 83.13(10)
Bpy-rings, 27.86(7)	N1-Ag1-O1w, 89.35(11)	O13-Ag1-O13′, 85.22(10)
py-Ag-py′, 7.19(10)		O13-Ag1-Ag1′, 113.22(7)

**Table 3:** Selected bond distances (Å) and angles (*º*) for compound **2**.

Hg1-N1a, 2.274(3)	N1a-Hg1-N1b, 114.74(11)	N1b-Hg1-O21, 115.85(11)
Hg1-N1b, 2.263(3)	N1a-Hg1-O11, 88.58(11)	O11-Hg1-O12, 53.82(11)
Hg1-O11, 2.490(3)	N1a-Hg1-O12, 140.71(12)	O11-Hg1-O21, 93.87(10)
Hg1-O12, 2.392(3)	N1a-Hg1-O21, 103.25(12)	O12-Hg1-O21, 91.53(11)
Hg1-O21, 2.286(3)	N1b-Hg1-O11, 134.38(12)	Bpy-rings, 30.43(16)
Hg1-O22, 2.762(3)	N1b-Hg1-O12 89.72(12)	py-Hg-py′, 30.43(16)
